# Pittsburg Dental Association

**Published:** 1880-01

**Authors:** W. F. Fundenberg, H. W. Orr

**Affiliations:** Pres.; Sec'y.


### ARTICLE VII.

### *Pittsburgh Dental Association.*

At a regular monthly meeting held June 10th, 1879, the following resolutions were unanimously adopted:

WHEREAS, The profession of Dentistry now suffers, and has suffered in the past from the empiricism of many of its practitioners, and

WHEREAS, The Dentist occupies the position of a public teacher his education should be such as to enable him to give a satisfactory and scientific reason for what he does, or proposes to do, therefore

*Resolved*, First, that an applicant for the position of student in the office of any member of this Association, shall be not less than eighteen years of age, of good moral character, and shall furnish satisfactory testimonials of the same.

*Second.*—A diploma from any Chartered Literary Institution, shall be deemed sufficient evidence of an applicant's preliminary educational qualifications.

*Third.*—The minimum preliminary educational qualifications shall be a thorough knowledge of Orthography, Reading, Writing, Arithmetic, Grammar and Geography, together with a knowledge of the Latin Grammar, and ability to read and translate a selection from any of the first five books of Cæsar's Commentaries.

*Fourth.*—That an Examining Board, composed of three active members of this Association, shall be elected at each annual meeting. The duty of said Board shall be to examine all applicants for admission, as students, to members of this Association. After examination of an applicant, each member of this Board will be entitled to ten votes, the applicant to receive twenty votes to entitle him to become a student.

W. F. FUNDENBERG, M. D., *Pres.*

H. W. ORR, D. D. S. *Sec'y.*

